# A Conceptual Approach to Partitioning a Vertical Profile of Phytoplankton Biomass Into Contributions From Two Communities

**DOI:** 10.1029/2021JC018195

**Published:** 2022-04-12

**Authors:** Robert J. W. Brewin, Giorgio Dall’Olmo, John Gittings, Xuerong Sun, Priscila K. Lange, Dionysios E. Raitsos, Heather A. Bouman, Ibrahim Hoteit, Jim Aiken, Shubha Sathyendranath

**Affiliations:** ^1^ Centre for Geography and Environmental Science College of Life and Environmental Sciences University of Exeter Cornwall UK; ^2^ Plymouth Marine Laboratory Plymouth UK; ^3^ National Centre for Earth Observation Plymouth Marine Laboratory Plymouth UK; ^4^ Program of Earth Science and Engineering King Abdullah University of Science and Technology Thuwal Saudi Arabia; ^5^ Department of Biology National and Kapodistrian University of Athens Athens Greece; ^6^ State Key Laboratory of Estuarine and Coastal Research East China Normal University Shanghai China; ^7^ Departamento de Meteorologia Universidade Federal do Rio de Janeiro (UFRJ) Rio de Janeiro Brazil; ^8^ Blue Marble Space Institute of Science (BMSIS) Seattle WA USA; ^9^ Department of Earth Sciences University of Oxford Oxford UK

**Keywords:** phytoplankton, community structure, vertical, ocean robotics, satellite, BGC‐Argo

## Abstract

We describe an approach to partition a vertical profile of chlorophyll‐a concentration into contributions from two communities of phytoplankton: one (community 1) that resides principally in the turbulent mixed‐layer of the upper ocean and is observable through satellite visible radiometry; the other (community 2) residing below the mixed‐layer, in a stably stratified environment, hidden from the eyes of the satellite. The approach is tuned to a time‐series of profiles from a Biogeochemical‐Argo float in the northern Red Sea, selected as its location transitions from a deep mixed layer in winter (characteristic of vertically well‐mixed systems) to a shallow mixed layer in the summer with a deep chlorophyll‐a maximum (characteristic of vertically stratified systems). The approach is extended to reproduce profiles of particle backscattering, by deriving the chlorophyll‐specific backscattering coefficients of the two communities and a background coefficient assumed to be dominated by non‐algal particles in the region. Analysis of the float data reveals contrasting phenology of the two communities, with community 1 blooming in winter and 2 in summer, community 1 negatively correlated with epipelagic stratification, and 2 positively correlated. We observe a dynamic chlorophyll‐specific backscattering coefficient for community 1 (stable for community 2), positively correlated with light in the mixed‐layer, suggesting seasonal changes in photoacclimation and/or taxonomic composition within community 1. The approach has the potential for monitoring vertical changes in epipelagic biogeography and for combining satellite and ocean robotic data to yield a three‐dimensional view of phytoplankton distribution.

## Key Points

- Gaussian and Sigmoid functions are used to partition vertical profiles of chlorophyll‐a concentration into contributions from two communities
- The approach is extended to the modeling of particle backscattering and applied to a Biogeochemical‐Argo float in the northern Red Sea
- The approach reveals contrasting phenology in the two communities of phytoplankton

## 1 Introduction

Phytoplankton are microscopic, single‐celled algae that live in the sunlit region of the ocean, the epipelagic zone, and perform photosynthesis. They are at the base of the marine food‐web, channeling energy from the sun into the marine ecosystem, where it propagates to higher trophic levels and to humans through seafood consumption. Phytoplankton, together with physical processes, modulate the CO_2_ concentration in the ocean, impacting air‐sea CO_2_ gas exchange, helping control the climate of our planet. Phytoplankton are considered an essential climate variable (GCOS, 2011), and monitoring their abundance at global scales is required for understanding and predicting the impact of climate change on marine ecosystems.

With an unparalleled capability to view the entire surface layer of the ocean within a few days and at synoptic scales, satellite remote sensing of ocean color is recognized as the main source of data for assessing changes in global phytoplankton abundance (Sathyendranath et al., 2017; Siegel & Franz, 2010). However, the ocean color signal retrieved passively by satellites is only representative of the surface layer (at most, and in the clearest waters, 50 m depth). In the seasonally and permanently stratified oceans (>70% of oceanic waters), there exists a community of phytoplankton below the surface layer (50–200 m depth) hidden from the eyes of the satellite (Cornec et al., 2021; Cullen, 1982, 2015). Monitoring full water column phytoplankton abundance synoptically requires integrating satellite data with measurements from deeper parts of the epipelagic. In the past, this has been done using ship‐based observations, collected from CTD rosette sampling. However, recent years have seen a proliferation in ocean robotic platforms that can sample the entire epipelagic with unprecedented coverage (Chai et al., 2020). Combining satellite and ocean robotic monitoring offers huge potential for understanding and predicting changes in total water column phytoplankton abundance (Claustre et al., 2020).

Combining satellite and in situ observations involves bridging the contrasting spatial and temporal scales of the measurements from the two systems: the in situ measurements, discretely distributed in space, and the synoptic data from satellites. Historically, empirical equations have been proposed to capture the vertical distribution of chlorophyll‐a concentration (a measure of phytoplankton biomass). The parameters of these equations, derived from fits to in situ profiles, are then related to some property of the surface ocean (e.g., trophic levels derived from chlorophyll‐a observations), or to time and space (e.g., biogeochemical provinces, seasons), such that parameters can be mapped over large scales, and functions used to extrapolate the surface fields seen from a satellite down through the entire epipelagic zone. These methods include Gaussian functions (e.g., Morel & Berthon, 1989; Platt & Sathyendranath, 1988; Uitz et al., 2006), sigmoid functions (e.g., Mignot et al., 2011), a combination of both functions (e.g., Carranza et al., 2018), and statistical methods (e.g., Sauzède et al., 2015). These approaches have proven useful for satellite models of ocean primary production (e.g., Antoine & Morel, 1996; Brewin et al., 2017; Longhurst et al., 1995; Platt & Sathyendranath, 1988; Sathyendranath et al., 1995; Uitz et al., 2010) and have been extended to other remotely‐sensed proxies of phytoplankton biomass, such as the particle backscattering coefficient (Sauzède et al., 2016).

Although the chlorophyll‐a concentration is used commonly as a measure of phytoplankton biomass, it can change independently in response to changing growth conditions (e.g., photoacclimation; Geider et al., 1996; Jackson et al., 2017). The particle backscattering coefficient is sensitive to both algal and non‐algal particles, though the extent of which is still an active area of research (Dall’Olmo et al., 2009; Koestner et al., 2020; Organelli et al., 2018, 2020; Stramski et al., 2004). The algal contribution to particle backscattering is thought to correlate with the biomass of phytoplankton in carbon units (Behrenfeld et al., 2005; Graff et al., 2015; Martínez‐Vicente et al., 2013). Variations in the vertical distribution of these two proxies of phytoplankton biomass (chlorophyll‐a and carbon) are thought to relate to photoacclimation processes or to shifts in phytoplankton composition (Cullen, 2015; Fennel & Boss, 2003).

Most empirical approaches describing vertical changes in phytoplankton deal with total phytoplankton. However, some methods have been extended further to partition a profile of total phytoplankton biomass into the contributions from different phytoplankton groups, partitioned according to size and/or taxa (e.g., Brewin et al., 2010, 2017; Rembauville et al., 2017; Sauzède et al., 2015; Uitz et al., 2006), a useful approach considering the differing roles phytoplankton groups have in ocean biogeochemical cycles (IOCCG, 2014; Le Quéré et al., 2005). One such approach, proposed by Lange et al. (2018), focused solely on the numerical abundance of cells of the phytoplankton species *Prochlorococcus*, partitions the vertical distribution of *Prochlorococcus* abundance into two populations, one that dominates the surface layer (high‐light adapted) and one the subsurface (low‐light adapted). This interesting approach considers, explicitly, vertical differences in growth environment and *Prochlorococcus* habitat within the epipelagic. The approach can be used for deriving the contribution from the surface population, seen directly by a satellite, to a vertical profile of *Prochlorococcus* cell abundance.

Here, we extend the concepts of Lange et al. (2018) to partition a vertical profile of chlorophyll‐a into two communities of phytoplankton, one that resides principally in the turbulent mixed‐layer and seen from space, the other below the mixed‐layer, not seen from space. The approach, which builds on earlier functions for describing the vertical distribution of chlorophyll‐a (sigmoid and Gaussian), is applied to a time‐series of profiles from a Biogeochemical‐Argo float in the northern Red Sea and is extended to reproduce profiles of particle backscattering, by considering the chlorophyll‐specific backscattering coefficients of the two communities and a background coefficient of non‐algal particles. The seasonal dynamics of the two communities are analyzed alongside environmental data and the approach is discussed in the context of vertical changes in epipelagic biogeography and of combining satellite and ocean robotic data.

## 2 Materials and Methods

A list of all symbols and definitions used in the paper is provided in Supplementary Table S1.

### 2.1 Study Site

**Figure 1:**
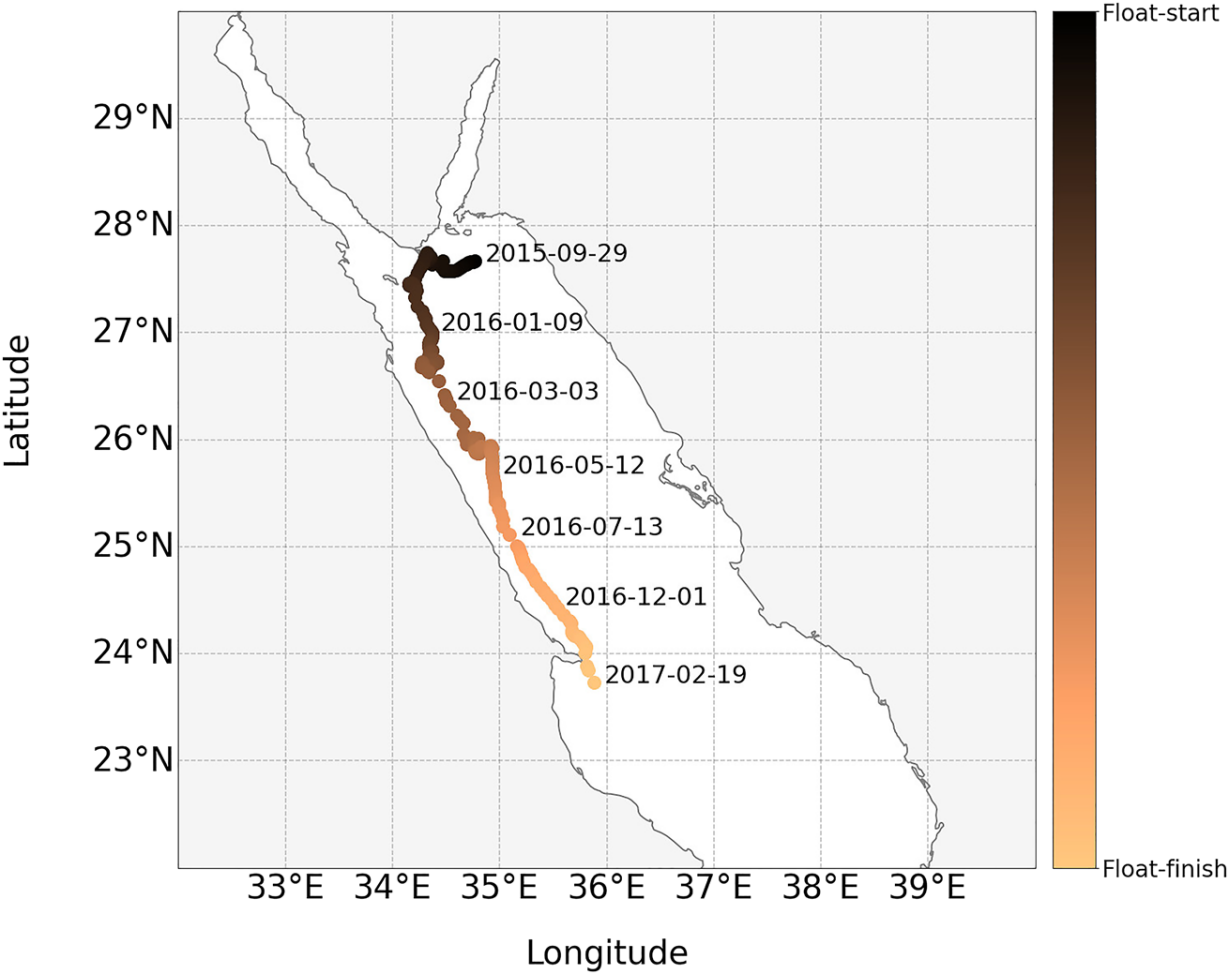
Map showing the locations of Biogeochemical Argo float profiles of over the life of the float (September 2015 to February 2017) in the northern Red Sea.

The chosen study site was the northern Red Sea (Figure 1). The Red Sea is a narrow, semi‐enclosed basin, connected to the Mediterranean Sea in the north through the Suez Canal, and to the Gulf of Aden and the Arabian Sea in the south, through the strait of Bab‐el‐Mandeb (Raitsos et al., 2013). It is the world's northernmost tropical sea and among the warmest and most saline (Belkin, 2009). It hosts one of the longest coral reef systems on Earth (Raitsos et al., 2017). The seasonal cycle of phytoplankton in the northern Red Sea is thought to be driven primarily by nutrient availability (Gittings et al., 2018; Papagiannopoulos et al., 2021). Higher phytoplankton abundance in winter is linked to increased convective mixing, driven by sea‐air heat exchange, that transports nutrients from deeper water into the surface layer (Acker et al., 2008; Papadopoulos et al., 2015; Triantafyllou et al., 2014; Yao et al., 2014). In summer, surface heating promotes stratification, reducing vertical mixing, lowering surface nutrients, and reducing surface phytoplankton abundance (Gittings, Raitsos, et al., 2019). During stratification, the presence of a deep chlorophyll‐a maximum at around 100 m has been observed (Gittings, Raitsos, et al., 2019; Kheireddine et al., 2017), a feature characteristic of many stratified regions (Cullen, 2015).

### 2.2 Data

Data collected from a Biogeochemical Argo (BGC‐Argo) float (Argo, 2020), in the northern Red Sea (WMO number 6901573) were acquired freely from the Ifremer Argo data assembly center (ftp://ftp.ifremer.fr/ifremer/argo/dac/coriolis/). This float was studied previously in Gittings, Raitsos, et al. (2019), and Kheireddine et al. (2020), includes profiles with a deep mixed layer (typical of vertically well‐mixed systems) and profiles with a deep chlorophyll‐a maximum (typical of stratified systems). Delayed mode profiles of pressure (dBar), temperature (°C), salinity (PSU), dissolved oxygen (*μ*mol kg^−3^), chlorophyll‐a (mg m^−3^), backscattering by particles (*b*_*bp*_ at the wavelength of 700 nm, m^−1^), and photosynthetically available radiation (PAR, *μ*mol quanta m^−2^ s^−1^), were used. For chlorophyll‐a, dissolved oxygen and *b*_*bp*_, adjusted profiles were used, which include additional processing steps as described in Schmechtig et al. (2014, 2019), to maximize the quality of the data and to correct for effects like non‐photochemical quenching for chlorophyll‐a (Bittig et al., 2019). A map of the geographical locations of the profiles from the BGC‐Argo float is provided in Figure 1, and additional details on the float are provided in Gittings, Raitsos, et al. (2019), and Kheireddine et al. (2020).

PAR data were converted from instantaneous measurements to daily integrals, by computing the day length as a function of time of year and latitude, scaling the profile given knowledge of the time of acquisition, and assuming a sinusoidal light cycle (Brewin et al., 2020). For profiles collected after sunset and before sunrise, data were disregarded. The diffuse attenuation coefficient for PAR (*K*_*d*_) was computed by fitting a Beer‐Lambert function to PAR data in the top 100 m of the water column. For the eight profiles where PAR was unavailable, *K*_*d*_ was estimated empirically from a linear fit (see Supplementary Figure S1a) between *K*_*d*_ and the average chlorophyll‐a concentration in the top 10 m of the water column (*B*_*s10*_), where *K*_*d*_ = 0.036*B*_*s10*_ + 0.049 (*r* = 0.79, *p* =<0.001). A linear, as opposed to a traditional non‐linear fit, was selected as the distribution of residuals (see Supplementary Figure S1b) was normal at a 2.5% significance level (Anderson‐Darling‐Test; Stephens [1974]).

The euphotic depth (*Z*_*p*_) was taken to be 4.6/*K*_*d*_, and the average PAR in the mixed‐layer, and between the mixed‐layer and euphotic depth, were computed. Pressure data were converted to depth, density was computed from temperature and salinity, and the Brunt–Väisälä buoyancy frequency was computed for each profile, using the Python seawater package (version‐3.3; Fofonoff & Millard, 1983; Gill, 1982; Jackett & Mcdougall, 1995; Millero & Poisson, 1981). The average Brunt–Väisälä buoyancy frequency above 6.9 optical depths (1.5 times the euphotic depth) was computed for each profile as an index of stratification within the epipelagic zone. The mixed layer depth (*Z*_*m*_) for each profile was computed using the method of Holte and Talley (2009), using their temperature algorithm (Python package https://github.com/garrettdreyfus/python-holteandtalley), and depth‐integration of variables was carried out using the trapezoid method (Ossendrijver, 2016).

### 2.3 Model Development

#### 2.3.1 Chlorophyll‐a Theoretical Framework

Building on the earlier works of Morel and Berthon (1989) and Uitz et al. (2006), we conduct model development in dimensionless space, albeit with slight differences in the manner in which we dimensionalize the chlorophyll‐a profile. We start by computing the dimensionless optical depth (*τ*) by multiplying the geometric depth (m) by *K*_*d*_ (m^−1^). The chlorophyll‐a concentration (*B*) at depth (*z*) is then normalized by the surface chlorophyll‐a concentration (*B*_*s*_), taken to be the median chlorophyll‐a in the first optical depth (having removed any cases of unrealistically low values <0.01 mg m^−3^). The choice to normalize the chlorophyll‐a profile to its surface concentration as in Brewin et al. (2017), rather than by its depth‐integrated concentration (Morel & Berthon, 1989; Uitz et al., 2006), means the profile is scaled directly to surface values also observable through satellites, and the set of equations used can be expressed in their simplest form (minimum amount of parameters), acknowledging that for in vivo fluorescence measurements, surface chlorophyll‐a concentrations can be subject to additional uncertainties related to non‐photochemical quenching.

Next, we consider the normalized chlorophyll‐a concentration ($B^{*}$) at a given optical depth (*τ*) as a combination of two communities of phytoplankton. Our definition of the term community follows that described by Begon et al. (1990), but focused solely on phytoplankton, as a group of species that occur together in space and time. This broad definition is flexible enough to encompass shifts in the taxonomic composition within a community, for example, due to seasonal variations in physical and chemical forcing. $B^{*}$ is expressed as

(1) $$B^{*}\left(\tau \right)=B_{1}^{*}\left(\tau \right)+B_{2}^{*}\left(\tau \right).$$

Community 1 $\left(B_{1}^{*}\right)$ represents a group of phytoplankton species that resides principally in the turbulent mixed‐layer of the surface ocean, at the interface between the ocean and atmosphere, is adapted to variations in light in the mixed‐layer, and represents the assemblage most influential in spectral variations of reflected light observed through satellite remote‐sensing of ocean color. We begin by modeling community 1 as a function of *τ* using a two‐parameter sigmoid function, such that

(2) $$B_{1}^{*}\left(\tau \right)=1-\frac{1}{1+\exp\left[-S_{1}\left(\tau -\tau_{1}\right)\right]},$$

where *S*_1_ represents the rate of change in $B_{1}^{*}$ with *τ*, and *τ*_1_ represents the mid‐point of the slope. As *τ* tends toward zero, Equation 2 can be expressed as

(3) $$B_{1}^{*}\left(\tau \to 0\right)=1-\frac{1}{1+\exp\left(S_{1}\tau_{1}\right)}.$$

The product *S*_1_ *τ*_1_ is hereafter denoted *P*_1_, such that Equation 2 can be arranged as follows:

(4) $$B_{1}^{*}\left(\tau \right)=1-\frac{1}{1+\exp\left[-\frac{P_{1}}{\tau_{1}}\left(\tau -\tau_{1}\right)\right]}.$$

Next, we make the assumption that $B_{1}^{*}$ tends to one as *τ* tends to zero, resulting in total dominance of community 1 at the very surface ocean. To do that, we introduce the constraint that *P*_1_ > 4.6 (i.e., community 1 is >99% of the total community as *τ* tends to zero, see Equation 3).

Community 2 $\left(B_{2}^{*}\right)$ represents a group of phytoplankton species that resides principally in a stable environment below the mixed layer, is adapted to low‐light conditions, is not observable through passive, satellite, remote‐sensing of ocean color (but for a few extreme cases, see Stramska & Stramski [2003]), and is characteristic of a phytoplankton community at the deep chlorophyll‐a maximum. We model $B_{2}^{*}$ as a function of *τ* using a Gaussian function, such that

(5) $$B_{2}^{*}\left(\tau \right)=B_{2,m}^{*}\;\exp\left[-{\left(\frac{\tau -\tau_{2}}{\sigma }\right)}^{2}\right],$$

where $B_{2,m}^{*}$ is the maximum of $B_{2}^{*}$, *τ*_2_ the dimensionless depth at which $B_{2,m}^{*}$ occurs, and *σ* the width of the $B_{2,m}^{*}$ peak. As *τ* tends toward zero, Equation 5 reduces to

(6) $$B_{2}^{*}\left(\tau \to 0\right)=B_{2,m}^{*}\;\exp\left[-{\left(\frac{\tau_{2}}{\sigma }\right)}^{2}\right].$$

Considering our earlier assumption, that as *τ* tends to zero $B_{1}^{*}$ tends to one, it follows that for the same condition $B_{2}^{*}$ should tend to zero. For cases where $B_{2,m}^{*}<80$, when *τ* tends to zero $B_{2}^{*}$ will be <1% if *τ*_2_ is higher than three *σ* (Equation 6). Combining Equations 4 and 5 we arrive at

(7) $$B^{*}\left(\tau \right)=1-\frac{1}{1+\exp\left[-\frac{P_{1}}{\tau_{1}}\left(\tau -\tau_{1}\right)\right]}+B_{2,m}^{*}\;\exp\left[-{\left(\frac{\tau -\tau_{2}}{\sigma }\right)}^{2}\right].$$

The total chlorophyll‐a concentration (*B*) at depth (*z*) can be reconstructed by scaling Equation 7 by *B*_*s*_ and *K*_*d*_. The scaled profile becomes equivalent to the Gaussian profile with a constant background introduced by Platt and Sathyendranath (1988) if the first term (community 1) is on the right‐hand side (the sigmoid function) is set as a constant. Here, the first term allows the chlorophyll‐a profile to approach zero at depth in the water column, which is not possible with a constant background. Our model requires two inputs (*B*_*s*_ and *K*_*d*_) and has five parameters (*P*_1_, *τ*_1_, $B_{2,m}^{*}$, *τ*_2_, and *σ*), the same number as that of other dimensionless models (e.g., Uitz et al., 2006). We note that this is not the first time a combined sigmoid and Gaussian function has been used to model the vertical distribution of chlorophyll‐a (see Carranza et al., 2018), but it is the first time, to our knowledge, that the two components are considered to represent two different communities.

We note that, for conditions where only community 1 is present, we drop the second term (community 2) on the right‐hand side of Equation 7 (the Gaussian function), such that

(8) $$B^{*}\left(\tau \right)=1-\frac{1}{1+\exp\left[-\frac{P_{1}}{\tau_{1}}\left(\tau -\tau_{1}\right)\right]}.$$

#### 2.3.2 Chlorophyll‐a Model Tuning

To fit the model to a dimensionless profile of chlorophyll‐a (with *N* > 6) we only selected data for <9.2 optical depths (twice the euphotic depth), for profiles where *Z*_*m*_ *K*_*d*_ < 9.2, otherwise full profiles were used. The tuning of the model followed a two‐step process.

##### 2.3.2.1 Step 1

In the first step, Equation 8 is fitted to the profile (Python function *minimize*, using Levenberg‐Marquardt method), with an initial guess of model parameters (*τ*_1_ = *Z*_*m*_ *K*_*d*_, and *P*_1_ = 9) and a lower and upper bound of 4.6 and 100 for *P*_1_ respectively, and a lower bound of zero for *τ*_1_. The fitting method used a bootstrap (1,000 iterations) and median parameters (with 2.5% and 97.5% confidence intervals) were extracted from each profile bootstrap. If Equation 8 explained >90% of the variance in the profile (squared Pearson correlation coefficient, *r* ^2^ > 0.9) then we assume community 1 dominates the profile (no community 2 present) and the profile can be described using Equation 8 and the parameters (*P*_1_ and *τ*_1_) retained from the fit. In other words, community 2 is assumed to be absent, unless they contribute at least 10% to the variability in the chlorophyll‐a profile.

##### 2.3.2.2 Step 2

For cases where Equation 8 explains <90% of the variance (*r* ^2^ < 0.9) in step 1, then we move to the second step of the tuning, which involves fitting Equation 7. In this step, *P*_1_ and *τ*_1_ are estimated empirically from the mixed‐layer depth (*Z*_*m*_). Using profiles from mixed waters (where *Z*_*p*_ < *Z*_*m*_) that passed step 1 (*r* ^2^ > 0.9), we found a significant relationship between *τ*_1_ and *Z*_*m*_ *K*_*d*_ (*τ*_1_ = 0.62*Z*_*m*_ *K*_*d*_ + 2.29, *r* = 0.78, *p* < 0.001, Supplementary Figure S2a), and a significant, albeit weaker, relationship between *P*_1_ and *τ*_1_ ($P_{1}=10^{0.08\tau_{1}+0.66}$, *r* = 0.42, *p* = 0.016, Supplementary Figure S2b). In this second step, *P*_1_ and *τ*_1_ were first derived using these relationships and then fixed when fitting Equation 7, to derive the parameters $B_{2,m}^{*}$, *τ*_2_ and *σ*.

To ensure community 2 tended close to zero at the surface (where *τ* tends to 0), in the fitting of Equation 7, *τ*_2_ was forced to be three *σ* or higher. $B_{2,m}^{*}$ was constrained to vary between 0 and 100 and *σ* to be greater than zero. The fitting of Equation 7 used the same Python packages, and used a bootstrap (1,000 iterations) method, with median parameters (with 2.5% and 97.5% confidence intervals) for $B_{2,m}^{*}$, *τ*_2_ and *σ* extracted from each profile bootstrap. The second step was only retained if the Akaike information criterion (AIC) of the step 2 fit (Equation 7) was lower than in step 1 (i.e., the fit using Equation 8). If it was not lower, we assume community 1 dominates the profile and community two is absent, such that $B^{*}$ follows Equation 8. We acknowledge that this method of model tuning may need to be adapted for different BGC‐Argo floats and datasets, and different regions. A flow diagram of the chlorophyll‐a model tuning is provided in Supplementary Figure S3.

#### 2.3.3 Particle Backscattering Theoretical Framework

As with the chlorophyll‐a profiles, we start by normalizing each particle backscattering profile at 700 nm (*b*_*bp*_) by the surface particle backscattering (*b*_*bp*,*s*_), taken to be the median *b*_*bp*_ in the first optical depth. We then consider the normalised particle backscattering $\left(b_{bp}^{*}\right)$ at any optical depth (*τ*) as a combination of the two assemblages of phytoplankton, and a background component, such that

(9) $$b_{bp}^{*}\left(\tau \right)=b_{bp,1}^{*}(\tau )+b_{bp,2}^{*}(\tau )+b_{bp,k}^{*},$$

where $b_{bp,1}^{*}$ and $b_{bp,2}^{*}$ are the surface normalised backscattering coefficients for community 1 and 2 respectively, and $b_{bp,k}^{*}$ is a surface normalized constant background. Next, we assume $b_{bp,1}^{*}$ and $b_{bp,2}^{*}$ can be tied to the dimensionless profile of chlorophyll‐a for each community, such that

(10) $$b_{bp}^{*}\left(\tau \right)=\omega_{1}B_{1}^{*}\left(\tau \right)+\omega_{2}B_{2}^{*}\left(\tau \right)+b_{bp,k}^{*},$$

where *ω*_1_ and *ω*_2_ are scaling factors linking the two communities of phytoplankton ($B_{1}^{*}$ and $B_{2}^{*}$) to the surface, normalised backscattering coefficients of community 1 and 2 respectively (i.e., $b_{bp,1}^{*}=\omega_{1}B_{1}^{*}$ and $b_{bp,2}^{*}=\omega_{2}B_{2}^{*}$). For cases where only community 1 is present, Equation 10 reduces to

(11) $$b_{bp}^{*}\left(\tau \right)=\omega_{1}B_{1}^{*}\left(\tau \right)+b_{bp,k}^{*}.$$

Considering that as *τ* tends to zero, $B_{2}^{*}$ tends to zero ($\omega_{2}B_{2}^{*}=0$, such that $\omega_{2}=0/B_{2}^{*}=0$), $B_{1}^{*}$ tends one (*ω*_1_ × 1 = *ω*_1_) and $b_{bp}^{*}$ tends one, and if we make the assumption $b_{bp,k}^{*}<1$ (i.e., constant background backscattering is not higher than surface *b*_*bp*_), both Equations 10 and 11 reduce to

(12) $$b_{bp}^{*}\left(\tau \to 0\right)=\omega_{1}+b_{bp,k}^{*}=1.$$

Accordingly, *ω*_1_ can be expressed as $1-b_{bp,k}^{*}$. Equations 10 and 11 can therefore be re‐written as

(13) $$b_{bp}^{*}\left(\tau \right)=\left(1-b_{bp,k}^{*}\right)B_{1}^{*}\left(\tau \right)+\omega_{2}B_{2}^{*}\left(\tau \right)+b_{bp,k}^{*},$$

and

(14) $$b_{bp}^{*}\left(\tau \right)=\left(1-b_{bp,k}^{*}\right)B_{1}^{*}\left(\tau \right)+b_{bp,k}^{*},$$

respectively.

Once *ω*_2_ and $b_{bp,k}^{*}$ are known, the chlorophyll‐specific backscattering coefficients of each community ($b_{bp,1}^{B}$ and $b_{bp,2}^{B}$) can be derived as

(15) $$b_{bp,1}^{B}=\left(1-b_{bp,k}^{*}\right)/\left(B_{s}/b_{bp,s}\right),$$

and

(16) $$b_{bp,2}^{B}=\omega_{2}/\left(B_{s}/b_{bp,s}\right),$$

where *B*_*s*_/*b*_*bp*,*s*_ is the ratio of surface chlorophyll‐a to surface particulate backscattering. These backscattering coefficients may be sensitive to the size and taxonomic composition of phytoplankton in each community (Brewin et al., 2012; Cetinić et al., 2015), and to their ratio of carbon to chlorophyll‐a, reflecting their photoacclimation status (Behrenfeld et al., 2005).

Finally, $b_{bp}^{k}$, a constant background particle backscattering coefficient, thought to be dominated by non‐algal particles in the region (Brewin et al., 2015; Kheireddine et al., 2021), can be computed as

(17) $$b_{bp}^{k}=b_{bp,k}^{*}b_{bp,s}.$$

The total backscattering coefficient can therefore be reconstructed as

(18) $$b_{bp}(z)=b_{bp,1}^{B}B_{1}(z)+b_{bp,2}^{B}B_{2}(z)+b_{bp}^{k}.$$

#### 2.3.4 Particle Backscattering Model Tuning

Having derived $B_{1}^{*}\left(\tau \right)$ and $B_{2}^{*}\left(\tau \right)$, the two key parameters *ω*_2_ and $b_{bp,k}^{*}$ required to solve Equations (9), (10), (11), (12), (13), (14), (15), (16), (17), (18) were derived by fitting Equation 13 (for conditions when both communities exist) or Equation 14 (for conditions when community 1 dominates) to profiles of $b_{bp}^{*}$ and *τ* (Python function *minimize*, using Levenberg‐Marquardt method). For the backscattering profiles (with *N* > 6) we only selected data <500 m depth. The initial guesses for $b_{bp,k}^{*}$ and *ω*_2_ were set to 0.2 and 0.3 respectively, and both were constrained to a lower limit of 0.01 and for $b_{bp,k}^{*}$ an upper limit of 0.95 (i.e., $b_{bp,k}^{*}$ was constrained to not contribute more than 95% of *b*_*bp*,*s*_). As with previous fits, we used a bootstrap (1,000 iterations) method, with median parameters (with 2.5% and 97.5% confidence intervals) extracted from each profile bootstrap.

**Figure 2:**
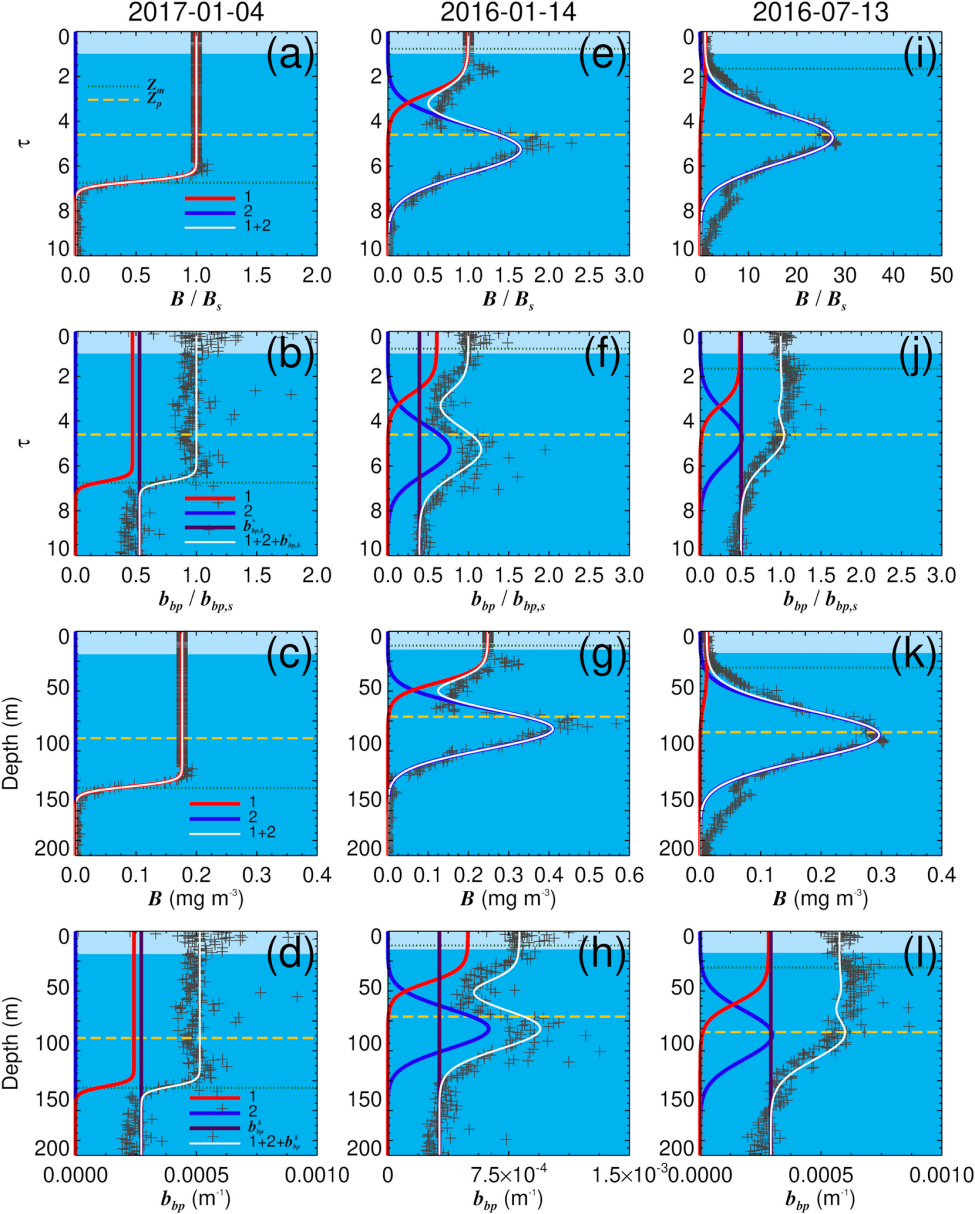
Examples of model fits to chlorophyll‐a (*B*) and particle backscattering (*b*_*bp*_) profiles from the biogeochemical Argo float; (a)–(d) are from a profile collected on the 4th of January 2017, where the water column is well mixed (*Z*_*m*_ > *Z*_*p*_); (e)–(f) are from a profile collected on the 14th January 2016, in stratified conditions (*Z*_*m*_ < *Z*_*p*_); (i)–(j) are from a profile collected on the 13th July 2016 in stratified conditions (*Z*_*m*_ < *Z*_*p*_). The dimensionless quantity *τ* represents the optical depth (geometric depth multiplied by diffuse attenuation coefficient), the line colors represent the community (red = community 1, blue = community 2, white = sum of components) and the background shading represents either the part of the water column seen by a passive ocean‐color satellite (light blue shading), or that below the eye of the satellite (darker blue shading).

Three examples of model fits to chlorophyll‐a and particle backscattering profiles from the BGC‐Argo float, one for a well‐mixed condition (where *Z*_*m*_ > *Z*_*p*_) and two for stratified conditions (where *Z*_*m*_ < *Z*_*p*_), are provided in Figure 2, and a sensitivity analysis of these model fits (varying model parameters and input) on the same three profiles is provided in Supplementary Figure S4. As in the chlorophyll‐a model, we acknowledge that this method of model tuning may need to be adapted for different BGC‐Argo floats and datasets, and for different regions of interest. An example of Jupyter Notebook Python Script, processing this BGC‐Argo float and tuning the models (without bootstrapping) is provided on this GitHub page (https://github.com/rjbrewin/Two-community-phyto-model) and details of how to run it without having to install software are provided as Supplementary Information to this manuscript.

### 2.4 Phenology Algorithm

Consistent with studies on phytoplankton phenology in the Red Sea using satellite remote‐sensing of ocean color and BGC‐Argo floats (Gittings, Raitsos, et al., 2019; Racault et al., 2015), we used a threshold method, based on cumulative sums of anomalies, to estimate the following phytoplankton phenology metrics of an annual time‐series: bloom initiation, termination, and duration. Firstly, a time‐series of column integrated chlorophyll‐a from the BGC‐Argo float (either from community 1 or 2) was linearly interpolated to a daily time step. The time‐series was then smoothed with a 15‐day filter (Savitzky‐Golay filter, using Python function scipy.signal.savgol_filter with the nearest mode). Next, and following Gittings, Raitsos, et al. (2019), we defined the threshold criterion as the median of the time series plus 5%, which was subtracted from the time‐series to derive a time‐series of anomalies. The cumulative sums of anomalies were then calculated, followed by the gradient in the cumulative sums, used to identify the timing of the transition between increasing and decreasing trends. The initiation of the bloom was identified as the period when the gradient of the time series first changed the sign to positive and remained positive for more than 15 days. Similarly, the termination of the bloom was identified when the gradient of the time series changed the sign to negative, following the initiation of the bloom, and remained negative for more than 15 days. Duration of the bloom was calculated as the time between initiation and termination. The annual period selected for the analysis varied between community 1 (October 2015 to October 2016) and community 2 (January 2016 to January 2017), to account for the fact that the peak timing of the communities (maximum chlorophyll‐a of each time series) was around 5 months apart, and to ensure the time‐series started during the lowest chlorophyll‐a period.

## 3 Results and Discussion

### 3.1 Seasonality in Physical and Biological Variables in the Northern Red Sea

Contour plots of all variables in the upper 200 m over the duration of the BGC‐Argo float (Supplementary Figure S5) illustrate a distinct seasonality in the northern Red Sea, consistent with previous studies (Acker et al., 2008; Gittings et al., 2018; Kheireddine et al., 2020; Papadopoulos et al., 2015; Raitsos et al., 2013; Sofianos & Johns, 2003; Yao et al., 2014). The winter period (November‐March) is characterized by deep vertical mixing, with lower average temperatures and higher salinity, and with chlorophyll‐a and *b*_*bp*_ more uniformly distributed. In the summer (May‐September), the water column becomes more stratified (higher Brunt–Väisälä buoyancy frequency index), light is more intense and penetrates further into the water column, and the chlorophyll‐a profile is characterized by the presence of a deep‐chlorophyll‐a maximum at around 100 m depth.

### 3.2 Chlorophyll‐a Model Results

**Figure 3:**
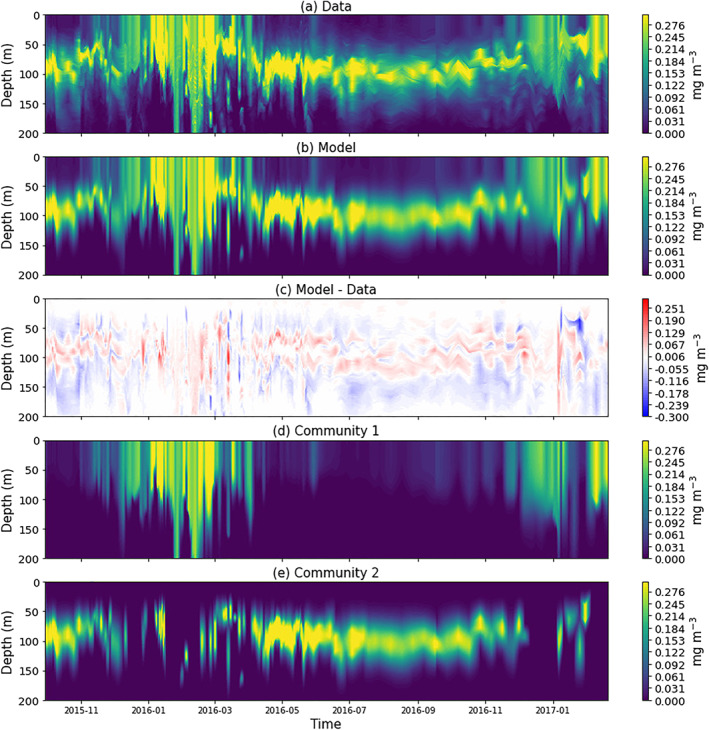
Contour plots of the chlorophyll‐a concentration over the duration of the Biogeochemical Argo (BGC‐Argo) float in the top 200 m of the water column. (a) Total chlorophyll‐a data from the BGC‐Argo float. (b) Model output of total chlorophyll‐a from tuning model to the data. (c) Differences in total chlorophyll‐a between model output and data. (d) Model output of chlorophyll‐a for community 1. (e) Model output of chlorophyll‐a for community 2.

In general, the model captures the patterns and dynamics in the data, with higher and more uniformly distributed chlorophyll‐a in winter, and a deep‐chlorophyll‐a maximum in summer (Figures 3a and 3b). The model has a slight tendency to underestimate chlorophyll‐a in deeper parts of the water column (Figure 3c, mean difference at 6.9 optical depths = −0.02 mg m^−3^), and slightly overestimates above and below the peak of the deep‐chlorophyll‐a maximum during summer months (Figure 3c, mean difference at the deep‐chlorophyll‐a maximum from June to August = 0.015 mg m^−3^). The model is not designed to capture rarer and more complex profiles (e.g., double deep‐chlorophyll‐a maximum, see Figure 3c February 2017). It is worth noting that during that period the float was close to the Ras Banas peninsula and coral reefs of Egypt (Figure 1), which may explain the complexity of some of the chlorophyll‐a profiles. Model parameters vary over the time‐series (see Supplementary Figure S6), reflecting seasonal changes in the shape of the chlorophyll‐a profile.

**Figure 4:**
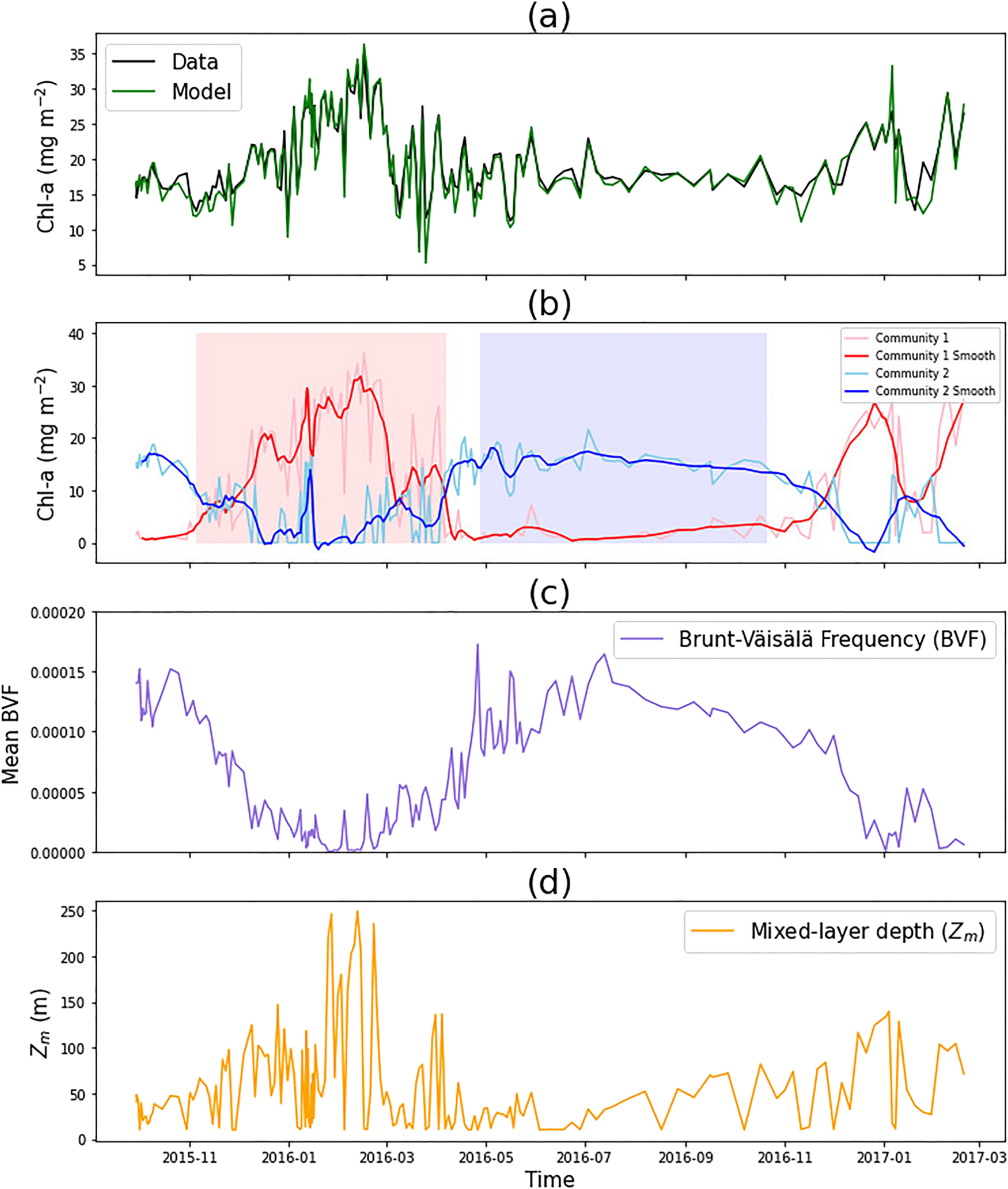
(a) Column integrated total chlorophyll‐a concentrations (down to an optical depth of 6.9) from the data and model over the duration of the Biogeochemical Argo (BGC‐Argo) float. (b) Column integrated chlorophyll‐a concentrations for the two communities of phytoplankton over the duration of the BGC‐Argo float (smoothed data were computed using Python function scipy.signal.medfilt, with a kernel size of 11 and nearest mode). Light red (blue) shaded background represents the phenology metrics for community 1 (community 2), representing initiation, duration and termination. (c) The average Brunt–Väisälä buoyancy frequency index in the top 6.9 optical depths over the duration of the BGC‐Argo float. (d) Mixed‐layer depth (*Z*_*m*_) over the duration of the BGC‐Argo float.

Figures 3d and 3e show the model output from the two communities of phytoplankton. Community 1 is seen to dominate during the winter period (November to April), whereas community 2 is more prominent during the summer, and with the exception of a few sporadic cases, is absent in the winter months. Figure 4 shows the column‐integrated chlorophyll‐a concentrations (down to an optical depth of 6.9, which is 1.5 times the euphotic depth, a boundary used in other studies (e.g., Brewin et al., 2017; Uitz et al., 2006)). Overall, the model is in good agreement with the data for total chlorophyll‐a (Figure 4a). The integrated chlorophyll‐a concentrations for the two communities reveal a contrasting pattern, with community 1 blooming in the winter (initiating in November, terminating in April, with a duration of around 5 months) and community 2 dominating in the summer (initiating in April, terminating in October, with a duration of around 6 months). Figures 4c and 4d show the average Brunt–Väisälä buoyancy frequency index in the top 6.9 optical depths (used as an index of stratification), and the mixed‐layer depth respectively. The integrated chlorophyll‐a concentration in community 1 is inversely correlated with the average Brunt–Väisälä buoyancy frequency index (*r* = −0.82, *p* < 0.001), and community 2 is weakly positively correlated (*r* = 0.46, *p* < 0.001).

The differences in the seasonal progression of the two communities can be explained conceptually from a bottom‐up (resource allocation) perspective. Community 1, present in the surface ocean, is likely not limited by light (the northern Red Sea is a tropical ocean with relatively high surface light all year round) but is limited by nutrients (characteristic of surface waters in many tropical regions). Community 2, on the other hand, is present in waters with higher nutrient concentrations (nearer the nutricline), but being far deeper in the water column, is likely limited by light availability. During winter, enhanced convective mixing (as illustrated by a deepening of the mixed layer, Figure 4d) pumps nutrients from depth into the surface mixed‐layer and ignites a bloom in community 1. This bloom reduces light availability below the mixed‐layer, limiting the growth of community 2. Alternatively, in summer, enhanced stratification promotes a shallow mixed‐layer, which reduces the availability of nutrients for community 1, limiting their growth. Higher surface light in summer, coupled with less shading of light by community 1 (owing to low concentrations) enhances light availability at depth and promotes the growth of community 2. This explanation provides a simple, conceptual, explanation for the patterns observed in the data. However, it neglects effects of top‐down control on the two communities (e.g., zooplankton grazing and/or viral lysis), changes in photoacclimation, aeolian nutrient input, nitrogen fixation, and does not consider shifts in taxonomic composition within the communities.

### 3.3 Particle Backscattering Model Results

**Figure 5:**
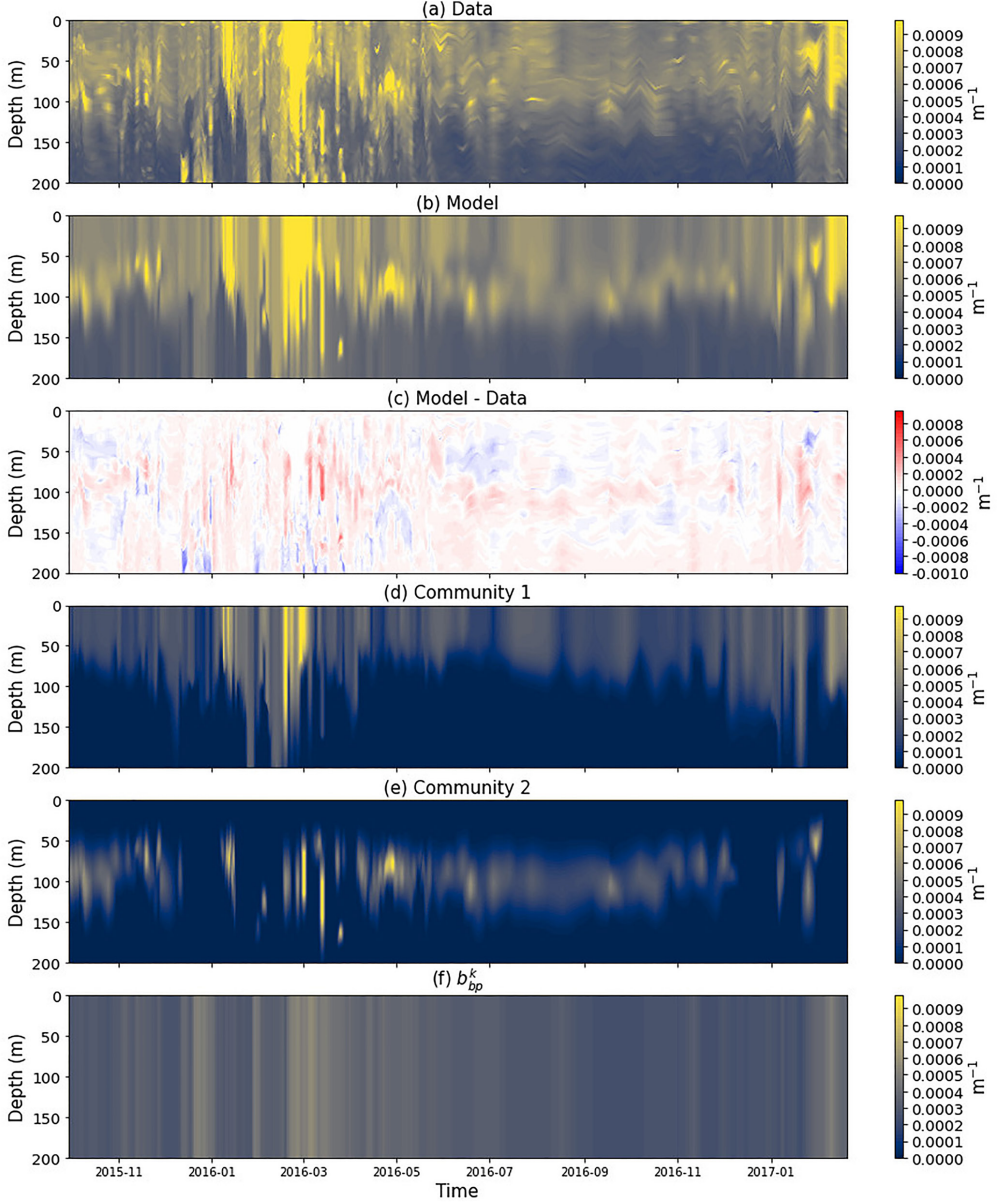
Contour plots of the *b*_*bp*_ over the duration of the Biogeochemical Argo (BGC‐Argo) float in the top 200 m of the water column. (a) Total *b*_*bp*_ from the BGC‐Argo float. Each profile was smoothed with a median filter (Python function scipy.signal.medfilt, with a kernel size of 11) to remove spikes in the data. (b) Model output of total *b*_*bp*_ from tuning model to the data. (c) Differences in total *b*_*bp*_ between model output and data (i.e., (b) − (a)). (d) Model output of *b*_*bp*_ for community 1. (e) Model output of *b*_*bp*_ for community 2. (f) The background backscattering coefficient $\left(b_{bp}^{k}\right)$ assumed to be associated with non‐algal particles in the region.

The model was found to capture seasonal variations in *b*_*bp*_ (Figures 5a–5c). The model fields are slightly smoother than the data (Figures 5a–5c), and not surprisingly considering the design of the model, it fails to capture occasional pulses of *b*_*bp*_ at depth during the winter months and spikes (which were removed from data in Figure 5a using a median filter). A deep‐particle maximum seems less of a prominent feature in the *b*_*bp*_ data fields during the summer months, in contrast to the deep chlorophyll‐a maximum (comparison between Figures 3 and 5), though perhaps a reflection of a higher presence of community 1 in the surface *b*_*bp*_ fields. The patterns in the two communities of phytoplankton (Figures 5d and 5e) are broadly consistent with the chlorophyll‐a model (Figures 3d and 3e), but the contribution to *b*_*bp*_ of community 1 is higher, and community 2 lower, as reflected by differences in the chl‐specific backscattering coefficients $\left(b_{bp}^{B}\right)$ of the two communities (see Supplementary Figure S6). This is broadly consistent with depth‐dependent variations in the carbon‐to‐chlorophyll‐a ratio of phytoplankton (Jackson et al., 2017; Sathyendranath et al., 2020). The backscattering coefficient has been used as a linear proxy for phytoplankton carbon (Behrenfeld et al., 2005; Bellacicco et al., 2018; Graff et al., 2015; Martínez‐Vicente et al., 2013) and $b_{bp}^{B}$ was found to be lower for community 2 (mean of 0.001 ± 0.0002) than community 1 (mean of 0.007 ± 0.001), most notably during summer months (see Supplementary Figure S6). This would suggest a lower carbon‐to‐chlorophyll‐a ratio for community 2, which is consistent with a theoretical understanding of low‐light adapted phytoplankton (Geider et al., 1998). If $b_{bp}^{B}$ reflects the photoacclimation status of the phytoplankton, we would expect a positive relationship with light availability, and in fact, $b_{bp,1}^{B}$ was found to be positively correlated with the average light in the mixed‐layer (Figure S7, *r* = 0.66, *p* < 0.001, statistics performed following log_10_‐transformation). On the other hand, $b_{bp,2}^{B}$ did not vary with light below the mixed layer and above the euphotic depth (Figure S7, *r* = 0.10, *p* = 0.270, statistics performed following log_10_‐transformation). These results suggest the model maybe useful for capturing variations in the photoacclimation status of the phytoplankton within community 1. This may even offer a route to deriving further information on the physiology of the phytoplankton in community 1, such as their photosynthetic rates, through models that link primary production and photoacclimation (e.g., Sathyendranath et al., 2020; Westberry et al., 2008). However, changes in $b_{bp}^{B}$ may also reflect shifts in the size or taxonomic composition of phytoplankton. For example, larger cells (e.g., diatoms) have been observed in waters with lower chl‐specific backscattering than those dominated by smaller phytoplankton (Brewin et al., 2012; Cetinić et al., 2015). In fact, seasonal shifts in the chl‐specific backscattering coefficient for community 1 (see Supplementary Figure S6) are consistent with satellite and in situ observations that show the presence of larger cells (likely prevalent in waters with low $b_{bp}^{B}$) during the winter bloom period and the presence of smaller phytoplankton (likely prevalent in waters with higher $b_{bp}^{B}$) during the summer in the northern Red Sea (Brewin et al., 2015; Gittings, Brewin, et al., 2019; Kheireddine et al., 2017; Mackey et al., 2007). Differences in the chl‐specific backscattering coefficient for community 1 and community 2 (Supplementary Figure S7) may also reflect depth variations in the size or taxonomic composition of phytoplankton.

Using a large dataset of scattering and absorption measurements collected in the Red Sea, Kheireddine et al. (2021) recently demonstrated that the background backscattering coefficient $\left(b_{bp}^{k}\right)$ is likely dominated by non‐algal particles. Our values of $b_{bp}^{k}$ were found to vary over the season, with a median value of 0.00035 m^−1^ (see Supplementary Figure S6), consistent with the ranges reported in Kheireddine et al. (2021) and globally by Bellacicco et al. (2019) at 700 nm. Higher $b_{bp}^{k}$ values were found during the winter period (Figure 5 and Supplementary Figure S6), possibly reflecting seasonal variation in the concentrations of non‐algal particles (Bellacicco et al., 2019, 2020). Nonetheless, the contribution of $b_{bp}^{k}$ to total *b*_*bp*_ was higher in the oligotrophic summer months, consistent with an increasing contribution of $b_{bp}^{k}$ to *b*_*bp*_ in oligotrophic conditions, as observed in other regions (Brewin et al., 2012; Zhang et al., 2020).

### 3.4 Limitations and Applications

The two‐step process used to tune the model to the BGC‐Argo float in the northern Red Sea worked well (Figures 3, 4, 5), but may need refining for other ocean regions. Other methods for tuning model parameters could be explored in the future, for example, using different minimizations schemes; refining the conditions set for the two step tuning; exploring single fit solutions without explicate thresholds; exploring different chi‐square functions (e.g., relative minimization rather than absolute); weighing the minimization with knowledge of data uncertainties; and exploring the use of other information on the BGC‐Argo float (e.g., floats with nutrient sensors) to aid model tuning.

Additional care and attention will need to be placed when fitting these models in regions with complex three‐dimensional dynamics, for example, in the presence of processes like eddy‐driven subduction (Llort et al., 2018). The BGC‐Argo float used here is considered to represent the broad region of interest (northern Red Sea). Whereas the float clearly captures the seasonal convective mixing characteristic of the region (Gittings et al., 2018; Papadopoulos et al., 2015), spatial variability within the region is known to exist, for example, through the transportation of water masses via eddies and surface currents (Gittings, Raitsos, et al., 2019). Such variability may explain some of the subtle changes seen over the duration of the float (Figures 1 and 3).

Despite the potential for data collection using autonomous platforms like BGC‐Argo, there are challenges to ensuring the data is of good quality. Chlorophyll‐a on BGC‐Argo floats is currently measured through in vivo fluorescence and converted using BGC‐Argo protocols that among other adjustments, correct for non‐photochemical quenching (Roesler et al., 2017; Schmechtig et al., 2014; Xing et al., 2012). Yet, this correction is notoriously challenging, and other issues can result in errors in the in vivo fluorescence estimates of chlorophyll‐a, considering the fluorescence yield can vary considerably between phytoplankton species and within a single species under contrasting environmental conditions (Cullen & Lewis, 1995; Kiefer, 1973a, 1973b; Slovacek & Bannister, 1973; Strickland, 1968). Whereas some confidence can be gained on the quality of the chlorophyll‐a from the BGC‐Argo float used here, considering values correspond well to high performance liquid chromatography (HPLC) pigment observations collected in the region (Kheireddine et al., 2017), and at the surface, to satellite estimates of chlorophyll‐a (Brewin et al., 2013, 2015; Gittings et al., 2019), one should be cautious of these issues when dealing with in vivo fluorescence data. The *b*_*bp*_ data from the BGC‐Argo float used here are in broad agreement with independent measurements collected in the region (Kheireddine et al., 2020, 2021), but challenges also exist in ensuring the quality of *b*_*bp*_ data on BGC‐Argo floats. For all BGC‐Argo data, it is important that rigorous, community‐assessed protocols are established and routinely updated (Claustre et al., 2020), and that efforts are placed on quantifying data uncertainties (Williams et al., 2017).

The model combines two existing empirical functions (sigmoid and Gaussian) commonly used for describing the vertical distribution of chlorophyll‐a (Mignot et al., 2011; Morel & Berthon, 1989; Platt & Sathyendranath, 1988; Uitz et al., 2006). This is not the first time these empirical functions have been combined for this purpose (see Carranza et al., 2018). However, rather than letting these functions fit the data freely, additional constraints were included to let the two functions represent two different communities of phytoplankton. A limitation to introducing these constraints is that the model has less freedom to fit the data. The method presented here was not developed with the objective of improving the fitting quality, but rather to build on existing functions (sigmoid and Gaussian) to track the surface and sub‐surface populations of phytoplankton independently of each other. The advantage of introducing these constraints is that we introduce a conceptual element to the fitting. It brings to the table an additional level of interpretation, of what phytoplankton the satellite can see (i.e., community 1 rather than community 2), and of vertical variations in phytoplankton community structure (Lange et al., 2018).

This conceptual approach can be used to investigate in greater detail, how surface phytoplankton phenology metrics derived from satellite observations are representative of column‐integrated phytoplankton phenology, building on the work of Gittings, Raitsos, et al. (2019). Separating the communities of phytoplankton within and below the mixed layer has the potential for improving our understanding of biogeochemical cycles in the ocean. For example, let us take the case of a permanently stratified region. Community 1 resides principally in the mixed‐layer, at the interface between the ocean and the atmosphere. Consequently, the fluxes of carbon, driven by the productivity of community 1, will be important in controlling exchanges of inorganic carbon between the ocean and the atmosphere. Community 2, situated below the mixed‐layer, likely supported by new nutrients, may contribute significantly to new production (Bouman et al., 2020). Trophic pathways within the marine ecosystem may vary between the two communities of phytoplankton, with implications for secondary production, trophic energy transfer, and even fisheries (Gittings et al., 2021). How these two communities of phytoplankton are changing has relevance to both ocean carbon and ocean health.

Another potential application of the model would be for extrapolating ship‐based observations (e.g., photosynthetic rates), collected at discrete depths (e.g., within the mixed layer and at the deep‐chlorophyll‐a maximum) to the full water column. For example, Tilstone et al. (2017) conducted photosynthesis‐irradiance experiments throughout the tropical Atlantic at two discrete depths (surface and at the deep‐chlorophyll‐a maximum). Marrying these observations with the model, and profiles of *b*_*bp*_ and chlorophyll‐a, could lead to the development of new models of primary production, that explicitly separate the contribution of the two communities of phytoplankton. The conceptual model presented here also suggests that an excellent strategy for sampling the water column for hard‐to‐measure properties, such as physiological rate parameters, would indeed be to sample the mixed layer and the deep chlorophyll‐a maximum, as a minimum requirement.

An inherent difficulty in studying oceanic biogeography, as compared with terrestrial biogeography, is the problem of observing 3D distributions that vary in both space and time (IOCCG, 2009). Although efforts have focused on mapping biogeography in deeper parts of the water column, such as the mesopelagic (Proud et al., 2017; Reygondeau et al., 2018; Sutton et al., 2017), the mapping of oceanic biogeography in the epipelagic zone has been driven primarily (though not exclusively) by surface measurements collected by satellite data (IOCCG, 2009; Longhurst, 2007; Reygondeau et al., 2013). Depth variations in biogeography within the epipelagic zone have not been studied in great detail. But now, with the advent of ocean robotic platforms such as BGC‐Argo floats, we are in a better position to do this and simple conceptual models like that presented here could be useful for mapping the habitats of the two communities of phytoplankton in space (3D) and time.

An additional benefit of the approach taken here is that it becomes feasible to reconcile vertical variations in two independent proxies of phytoplankton biomass, chlorophyll‐a and *b*_*bp*_. Recently, Cornec et al. (2021) used a global database of chlorophyll‐a and *b*_*bp*_ profiles, collected using BGC‐Argo floats, to describe spatial and temporal patterns in two types of deep chlorophyll‐a maximum profiles in the world's ocean, a carbon biomass maximum (deep biomass maximum), and a chlorophyll‐a maximum developed as a consequence of photoacclimation processes (deep photoacclimation maximum). The conceptual approach used here may offer further insight into the processes that control these types of profiles. For example, $b_{bp,2}^{B}$ was found to be relatively stable over the time‐series of the BGC‐Argo float studied here (Supplementary Figure S6) and did not vary with light availability (Supplementary Figure S7). However, $b_{bp,1}^{B}$ was highly variable (Supplementary Figure S6) and positively correlated with light availability (Supplementary Figure S7). It maybe that shifts between the two types of profiles are primarily a consequence of photoacclimation (and/or shifts in taxonomic structure) occurring in community 1, rather than any change in acclimation (and/or shifts in taxonomic structure) in community 2 at the deep chlorophyll‐a maximum.

A major driver for developing an approach like that presented here, that describes the vertical distribution of chlorophyll‐a and *b*_*bp*_, has been to bridge the contrasting temporal and spatial scales of surface satellite observations (high temporal frequency and spatial coverage) and subsurface in situ observations (low temporal frequency and spatial coverage), to produce four‐dimensional fields of chlorophyll‐a and *b*_*bp*_. The approach presented here is well suited for such extrapolations, with model functions scaled to three ocean‐color products routinely provided by space agencies (*b*_*bp*,*s*_, *B*_*s*_ (ratio of the two being $b_{bp,1}^{B}$), and *K*_*d*_). Other inputs, such as the mixed‐layer depth maps (*Z*_*m*_), can be acquired from models or observations. The parameters not directly available ($B_{2,m}^{*}$, *τ*_2_, *σ*, and $b_{bp,2}^{B}$) can be mapped by fitting our functions to in situ observations, then relating these parameters to some property of the surface ocean (e.g., trophic levels), or physical observations (e.g., the larger Argo array), or to time and space (e.g., biogeochemical provinces, seasons), such that they can be mapped over large scales, and used with the other inputs and parameters to extrapolate the surface fields seen from a satellite down through the epipelagic zone. As we move into an era of ocean robotic platforms, with an expanding number of in situ observations, model parameters can be mapped with a higher degree of confidence, and we can continue to improve our capability to monitor ocean biogeochemical cycles (Brewin et al., 2021; Claustre et al., 2021).

### 3.5 On the Two Communities of Phytoplankton

The concept of partitioning a profile of phytoplankton biomass into two communities stemmed from the consideration of vertical variability in environmental growth conditions. At the surface ocean, there exists a turbulent mixed layer, exposed to a fluctuating light and nutrient environment. Below this surface layer, and under conditions of high stratification, there exists a stable (low turbulence), low‐light environment, replete in nutrients and suitable for phytoplankton growth. The assumption we make is that these contrasting growth environments will promote different communities of phytoplankton. In the Red Sea, this assumption is supported by HPLC pigment data, showing vertical gradients in pigment composition (Kheireddine et al., 2017), flow cytometry, which has revealed vertical changes in picophytoplankton composition and abundance (Al‐Otaibi et al., 2020; Veldhuis & Kraay, 1993), and molecular methods, that have revealed vertical variations in both the eukaryotic and prokaryotic plankton communities and ecotypes (Fuller et al., 2005; Pearman et al., 2016, 2017; Shibl et al., 2014, 2016). Yet, despite increasing scientific interest in the Red Sea (Hoteit et al., 2021), it still remains a relatively under‐explored ecosystem, and more research is required to determine the exact composition of these phytoplankton communities identified and their seasonality.

We acknowledge our partitioning of phytoplankton into two communities is a simplification of their diversity. There will be environmental variations within each of these habitats that will promote shifts in the size and taxonomic structure of phytoplankton. Furthermore, as one transition from a stratified profile with the presence of both communities, to a situation where only one community exists, for example, due to the sudden deepening of the mixed layer (e.g., from sporadic events like the passage of a storm), phytoplankton from community 2 will inevitably be mixed into community 1. In other words, there will inevitably be exchanges between the two communities of phytoplankton. The time‐scales of competitive exclusion will dictate the point at which the natural phytoplankton composition will resume.

The application of our model to data in the northern Red Sea has provided some interesting insights into how a tropical ocean may change. Studies have suggested that climate change will enhance ocean stratification in tropical seas, reducing phytoplankton biomass (Doney, 2006). Our results in the northern Red Sea suggest community 1 is inversely correlated with stratification and community 2 is positively correlated (Figure 4). This finding raises the possibility that we might instead see a restructuring of phytoplankton biomass within the water column, favoring community 2. If that is the case, what impact might there be on the marine ecosystem? The model is designed to help address such questions.

## 4 Summary

Using two established empirical functions (sigmoid and Gaussian) for describing the vertical distribution of phytoplankton biomass, we developed an approach to partition a vertical chlorophyll‐a profile into two communities of phytoplankton: one present in the surface mixed‐layer of the ocean (community 1), and the other, below the mixed‐layer, in a stable, low‐light environment (community 2). The approach is tuned to a time‐series of chlorophyll‐a profiles collected by a BGC‐Argo float in the northern Red Sea and extended to reproduce profiles of particle backscattering, by deriving the chlorophyll‐specific backscattering coefficients of the two communities and a background coefficient related to non‐algal particles. Analysis of the time‐series reveals contrasting phenology metrics of the two communities, with community 1 dominating in winter and 2 in summer. We observed an inverse relationship between community 1 and stratification and a positive relationship for community 2. The chlorophyll‐specific backscattering coefficient for community 2 was found to be relatively stable over the time‐series, but that of community 1, highly variable, suggesting seasonal changes in photoacclimation and/or taxonomic composition within community 1. The approach would be useful for combining satellite and ocean robotic data, mapping vertical epipelagic biogeography, and for understanding the impact of climate change on phytoplankton biomass, with consequences for ocean biogeochemical cycles.

## Supporting information

Supporting Information S1

## Data Availability

These data were collected and made freely available by the International Argo Program and the national programs that contribute to it (https://argo.ucsd.edu, https://www.ocean-ops.org). The Argo Program is part of the Global Ocean Observing System. All data and code used in the paper are provided openly on a GitHub page (https://github.com/rjbrewin/Two-community-phyto-model). This includes an example Jupyter Notebook Python Script, processing this BGC‐Argo float and tuning the models. Details of how to run it without having to install software are provided as Supplementary Material to this manuscript.
