# Supplementary material for: A Conceptual Approach to Partitioning a Vertical Profile of Phytoplankton Biomass Into Contributions From Two Communities
Source: J Geophys Res Oceans. 2022 Apr 12;127(4):e2021JC018195. doi: 10.1029/2021JC018195 (PMC9285788; doi:10.1029/2021JC018195)
Supplement: Supplementary file 1 — Supporting Information S1 [file JGRC-127-0-s001.pdf]

Supplementary material for

A conceptual approach to partitioning a vertical profile of phytoplankton biomass into contributions from two communities

Robert J.W. Brewin[a], Giorgio Dall'Olmo[b,c], John Gittings[d,e], Xuerong Sun[a,f], Priscila K. Lange[g,h], Dionysios E. Raitsos[e], Heather A. Bouman[i], Ibrahim Hoteit[d], Jim Aiken[b], Shubha Sathyendranath[b,c]

[a]Centre for Geography and Environmental Science, College of Life and Environmental Sciences, University of Exeter, Penryn, Cornwall, United Kingdom
[b]Plymouth Marine Laboratory (PML), Prospect Place, The Hoe, Plymouth PL1 3DH, UK
[c]National Centre for Earth Observation, PML, Plymouth PL1 3DH, UK
[d]Program of Earth Science and Engineering, King Abdullah University of Science and Technology, Thuwal 23955-6900, Saudi Arabia
[e]Department of Biology, National and Kapodistrian University of Athens, Athens, Greece
[f]State Key Laboratory of Estuarine and Coastal Research, East China Normal University, Shanghai, China
[g]Departamento de Meteorologia, Universidade Federal do Rio de Janeiro (UFRJ), Rio de Janeiro, Brazil
[h]Blue Marble Space Institute of Science (BMSIS), Seattle, United States
[i]Department of Earth Sciences, University of Oxford, Oxford, United Kingdom

Journal of Geophysical Research, Oceans, 2022

1. Introduction

This supplementary material contains the follow Tables and Figures.

- Table S1. Symbols and definitions used in the paper.
- Figure S1. (a) K_d as a function of surface chlorophyll-a (B_{s10}) with a linear fit applied. (b) Residuals between modelled K_d (linear fit) and data.
- Figure S2. (a) The relationship between model parameter τ_1 and $Z_m K_d$ (mixed-layer depth multiplied by K_d) for profiles where $Z_m > Z_p$ (i.e. fully mixed euphotic zone), and where Eq. 8 in the paper explains >90 % of the variance in the profile ($r^2 > 0.9$). (b) The relationship between model parameters P_1 and τ_1 for the same set of profiles as (a).
- Figure S3. Flow diagram of the chlorophyll-a model tuning.

- Figure S4. Sensitivity analysis of model fits to three chlorophyll-a (B) and particle backscattering (b_{bp}) profiles from the BGC-Argo float. (a-b) fits to a profile collected on the 4th of January 2017 where the water column is well mixed ($Z_m > Z_p$), (c-d) fits to a profile collected on the 14th January 2016 in stratified conditions ($Z_m < Z_p$), and (e-f) fits to a profile collected on the 13th July 2016 in stratified conditions ($Z_m < Z_p$). Solid lines represent each component (red = community 1, blue = community 2, purple = b_{bp}^{k}), and lighter shading for each community and b_{bp}^{k} represent the minimum and maximum from an ensemble of simulations where all parameters and inputs were varied between assigned upper and lower boundaries, in every possible permutation. Upper and lower boundaries for inputs for B_s, K_d and Z_m were varied ± 10 % of their input values, and $b_{bp,s}$ was varied ± the standard deviation of $b_{bp,s}$ in the 1st optical depth. All other parameters were varied between confidence intervals derived from the bootstrapped fit, or from associated fitted functions (e.g. Fig. S2).

- Figure S5. Contour plots of all variables over the duration of the BGC-Argo float in the top 200 m of the water column. For b_{bp} each profile was smoothed with a median filter (Python function scipy.signal.medfilt, with a kernel size of 11) to remove spikes.

- Figure S6. Parameters of the model over the duration of the BGC-Argo float derived from tuning the model to chlorophyll-a and b_{bp} data.

- Figure S7. Relationships between the chlorophyll-specific backscattering coefficient for community 1 ($b_{bp,1}^{B}$) and the average light in the mixed-layer (cyan points), and the chlorophyll-specific backscattering coefficient for community 2 ($b_{bp,2}^{B}$) and the average light below the mixed layer and above the euphotic depth (red points).

An example Jupyter Notebook Python Script, processing this BGC-Argo float and tuning the models (without bootstrapping) is provided on this GitHub page (`https://github.com/rjbrewin/Two-community-phyto-model`). To run the script without having to install software go onto the GitHub page above, then click on the "launch binder" icon at the bottom of the README.md file. This will launch the notebook in binder. Once loaded (can take a minute or two), click on the "Example_fits_for_2_community_vertical_model.ipynb" file and the notebook will appear. You can then work through the notebook to see how the model is fitted in Python.

Table S1: Symbols and definitions used in the paper.

Symbol	Definition	Units
b_{bp}	The backscattering coefficient of particles	m^{-1}
$b_{bp,1}$	The community 1 backscattering coefficient of particles	m^{-1}
$b_{bp,2}$	The community 2 backscattering coefficient of particles	m^{-1}
$b_{bp,s}$	The surface backscattering coefficient of particles, the median b_{bp} in the 1st optical depth	m^{-1}
b^*_{bp}	The backscattering coefficient of particles normalised by its surface value ($b_{bp}/b_{bp,s}$)	dimensionless
$b^*_{bp,1}$	The community 1 backscattering coefficient of particles normalised by $b_{bp,s}$ ($b_{bp,1}/b_{bp,s}$)	dimensionless
$b^*_{bp,2}$	The community 2 backscattering coefficient of particles normalised by $b_{bp,s}$ ($b_{bp,2}/b_{bp,s}$)	dimensionless
$b^*_{bp,k}$	A constant background particle backscattering coefficient normalised by $b_{bp,s}$ ($b^k_{bp}/b_{bp,s}$)	dimensionless
$b^B_{bp,1}$	The chlorophyll-specific backscattering coefficients of community 1	m^2 [mg B]$^{-1}$
$b^B_{bp,2}$	The chlorophyll-specific backscattering coefficients of community 2	m^2 [mg B]$^{-1}$
b^k_{bp}	A constant background b_{bp}, thought to be dominated by non-algal particles	m^{-1}
B	The total chlorophyll-a concentration	$mg\,m^{-3}$
B_1	The chlorophyll-a concentration of community 1	$mg\,m^{-3}$
B_2	The chlorophyll-a concentration of community 2	$mg\,m^{-3}$
B_s	The surface total chlorophyll-a concentration, the median B in the 1st optical depth	$mg\,m^{-3}$
B_{s10}	The surface total chlorophyll-a concentration, the average in the top 10 m of the water column	$mg\,m^{-3}$
B^*	The chlorophyll-a concentration normalised by its surface value (B/B_s)	dimensionless
B^*_1	The chlorophyll-a concentration of community 1 normalised by surface total chlorophyll-a (B_1/B_s)	dimensionless
B^*_2	The chlorophyll-a concentration of community 2 normalised by surface total chlorophyll-a (B_2/B_s)	dimensionless
$B^*_{2,m}$	The maximum of B^*_2	dimensionless
K_d	The diffuse attenuation coefficient for Photosynthetically Available Radiation (PAR)	m^{-1}
p	Two-tailed p-value	dimensionless
P_1	The product of $S_1\tau_1$	dimensionless
PAR	Photosynthetically Available Radiation	μmol quanta $m^{-2}\,d^{-1}$
r	Pearson correlation coefficient	dimensionless
r^2	Squared Pearson correlation coefficient	dimensionless
S_1	The rate of change in B^*_1 with τ	dimensionless
z	Geometric depth	m
Z_m	Mixed-layer depth	m
Z_p	Euphotic depth	m
ω_1	Scaling factor linking B^*_1 to $b^*_{bp,1}$	dimensionless
ω_2	Scaling factor linking B^*_2 to $b^*_{bp,2}$	dimensionless
σ	The width of the $B^*_{2,m}$ peak	dimensionless
τ	The optical depth ($K_d z$)	dimensionless
τ_1	The mid-point of S_1 along the τ axis	dimensionless
τ_2	The dimensionless depth at which $B^*_{2,m}$ occurs	dimensionless

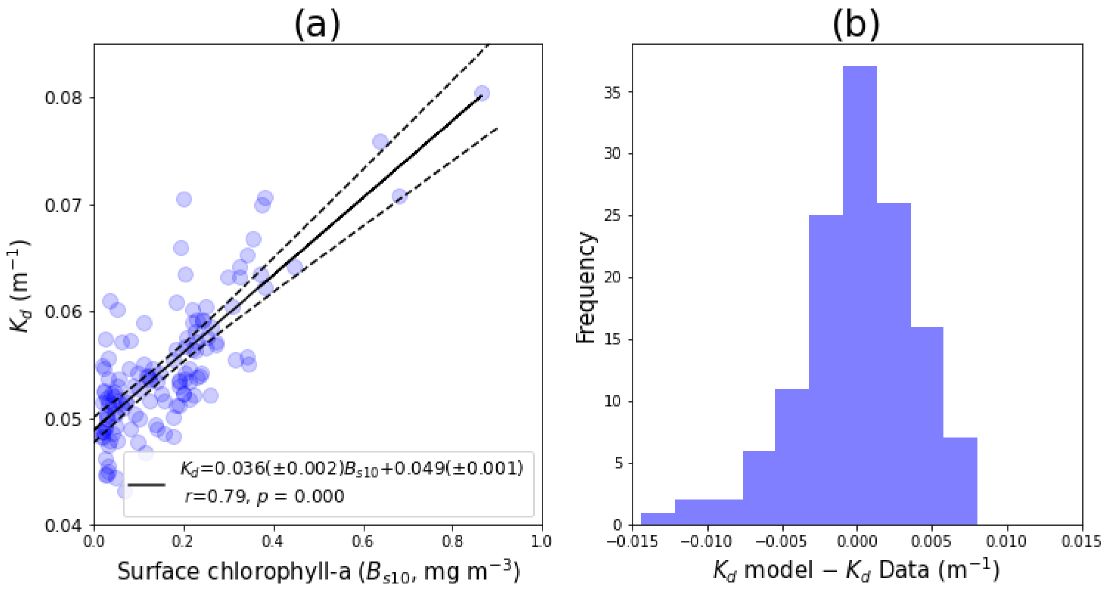

Figure S1: (a) K_d as a function of surface chlorophyll-a (B_{s10}) with a linear fit applied. (b) Residuals between modelled K_d (linear fit) and data.

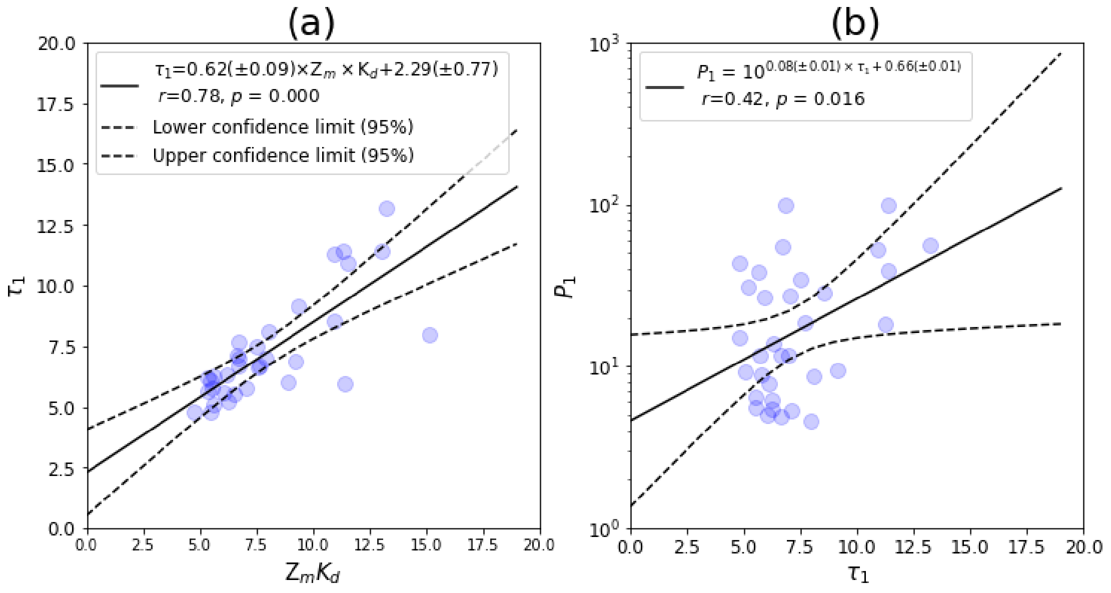

Figure S2: (a) The relationship between model parameter τ_1 and Z_mK_d (mixed-layer depth multiplied by K_d) for profiles where $Z_m > Z_p$ (i.e. fully mixed euphotic zone), and where Eq. 8 in the paper explains >90 % of the variance in the profile ($r^2 > 0.9$). (b) The relationship between model parameters P_1 and τ_1 for the same set of profiles as (a).

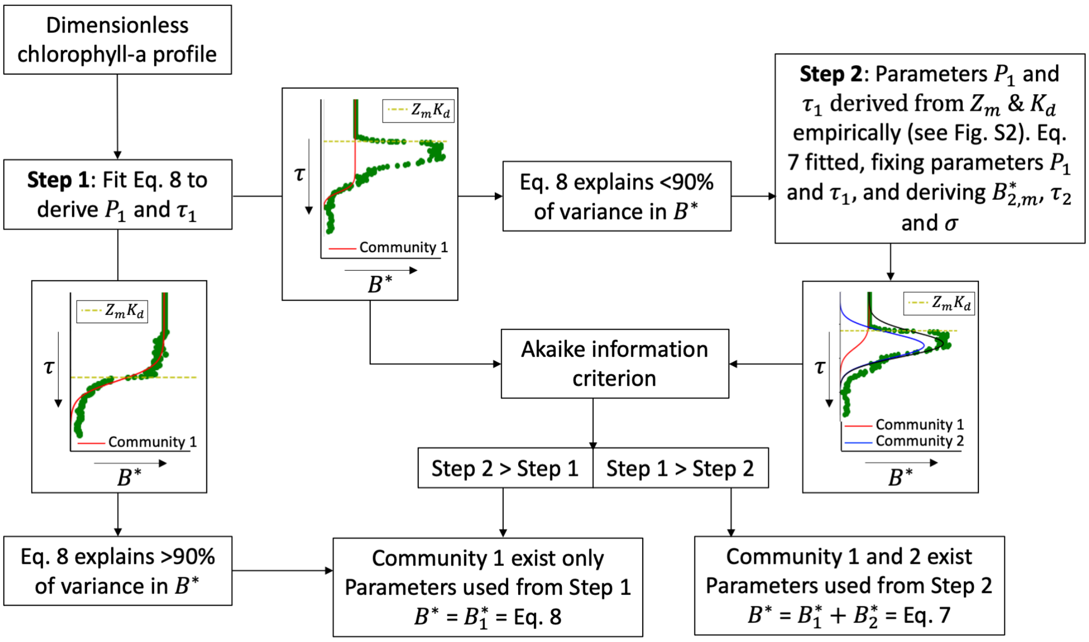

Figure S3: Flow diagram of the chlorophyll-a model tuning.

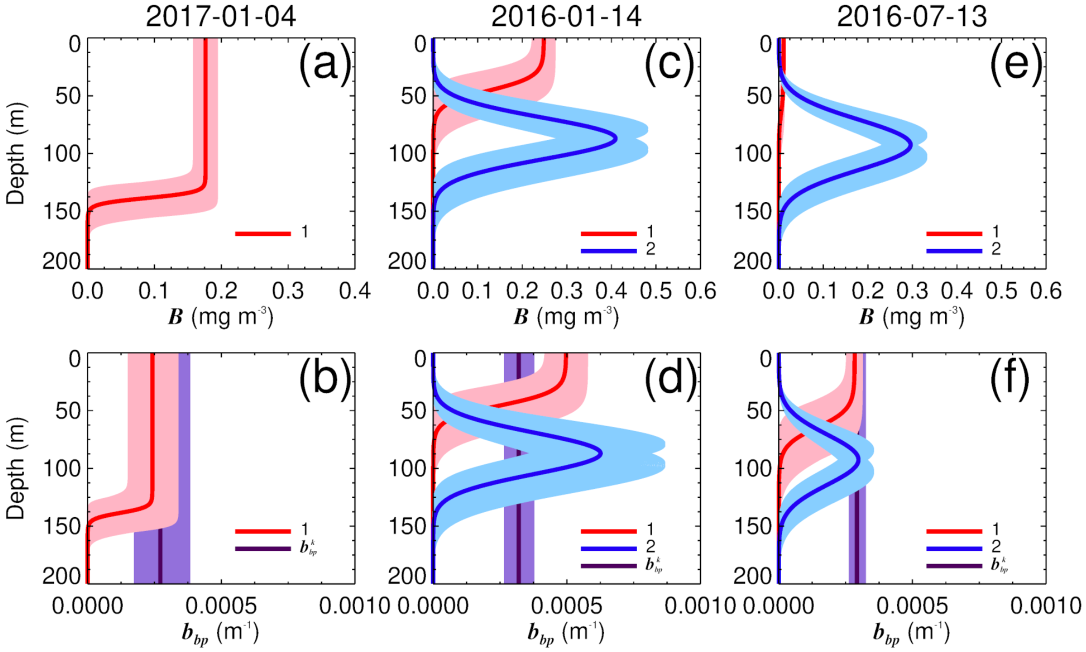

Figure S4: Sensitivity analysis of model fits to three chlorophyll-a (B) and particle backscattering (b_{bp}) profiles from the BGC-Argo float. (a-b) fits to a profile collected on the 4th of January 2017 where the water column is well mixed ($Z_m > Z_p$), (c-d) fits to a profile collected on the 14th January 2016 in stratified conditions ($Z_m < Z_p$), and (e-f) fits to a profile collected on the 13th July 2016 in stratified conditions ($Z_m < Z_p$). Solid lines represent each component (red = community 1, blue = community 2, purple = b_{bp}^k), and lighter shading for each community and b_{bp}^k represent the minimum and maximum from an ensemble of simulations where all parameters and inputs were varied between assigned upper and lower boundaries, in every possible permutation. Upper and lower boundaries for inputs for B_s, K_d and Z_m were varied $\pm$ 10 % of their input values, and $b_{bp,s}$ was varied $\pm$ the standard deviation of $b_{bp,s}$ in the 1st optical depth. All other parameters were varied between confidence intervals derived from the bootstrapped fit, or from associated fitted functions (e.g. Fig. S2).

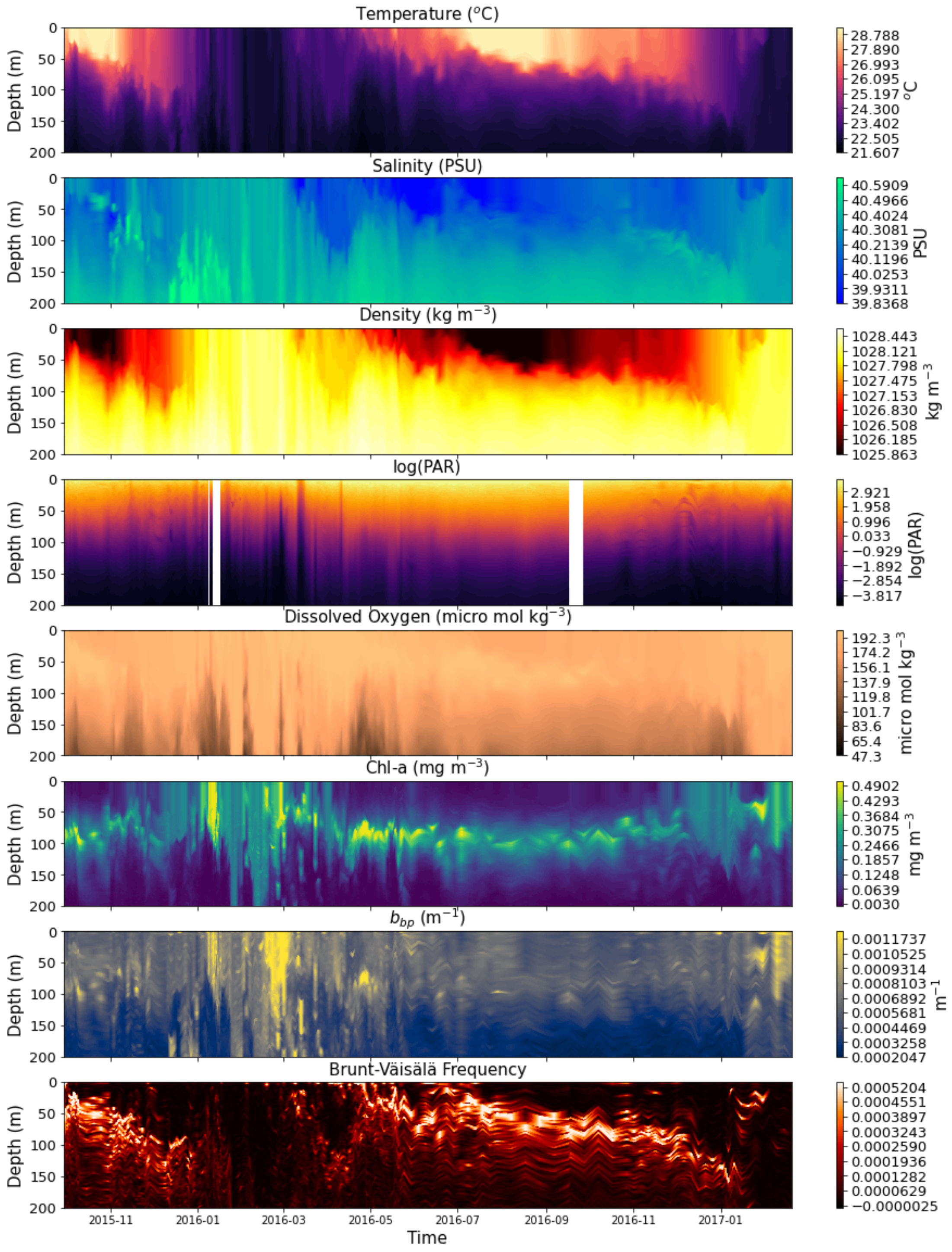

Figure S5: Contour plots of all variables over the duration of the BGC-Argo float in the top 200 m of the water column. For b_{bp} each profile was smoothed with a median filter (Python function scipy.signal.medfilt, with a kernel size of 11) to remove spikes.

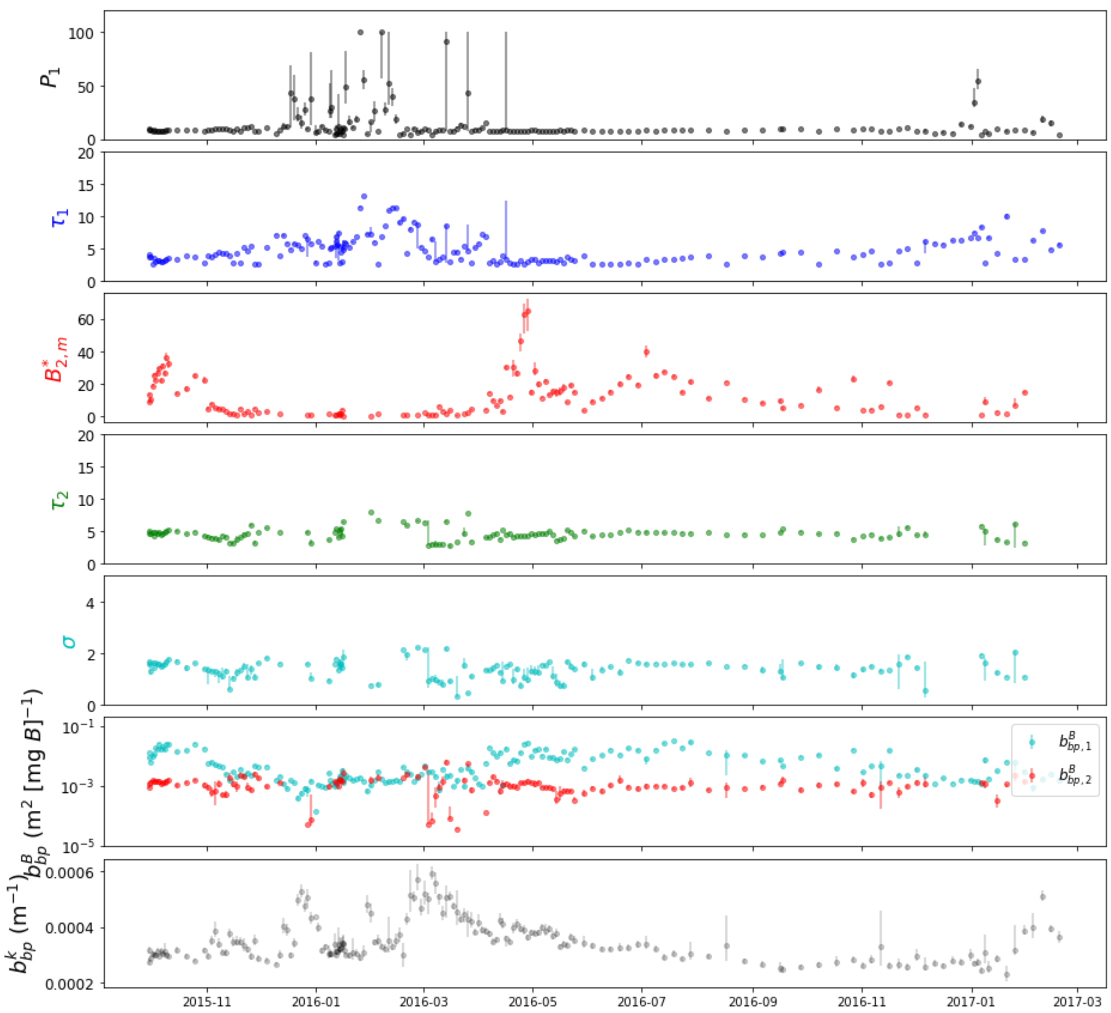

Figure S6: Parameters of the model over the duration of the BGC-Argo float derived from tuning the model to chlorophyll-a and b_{bp} data.

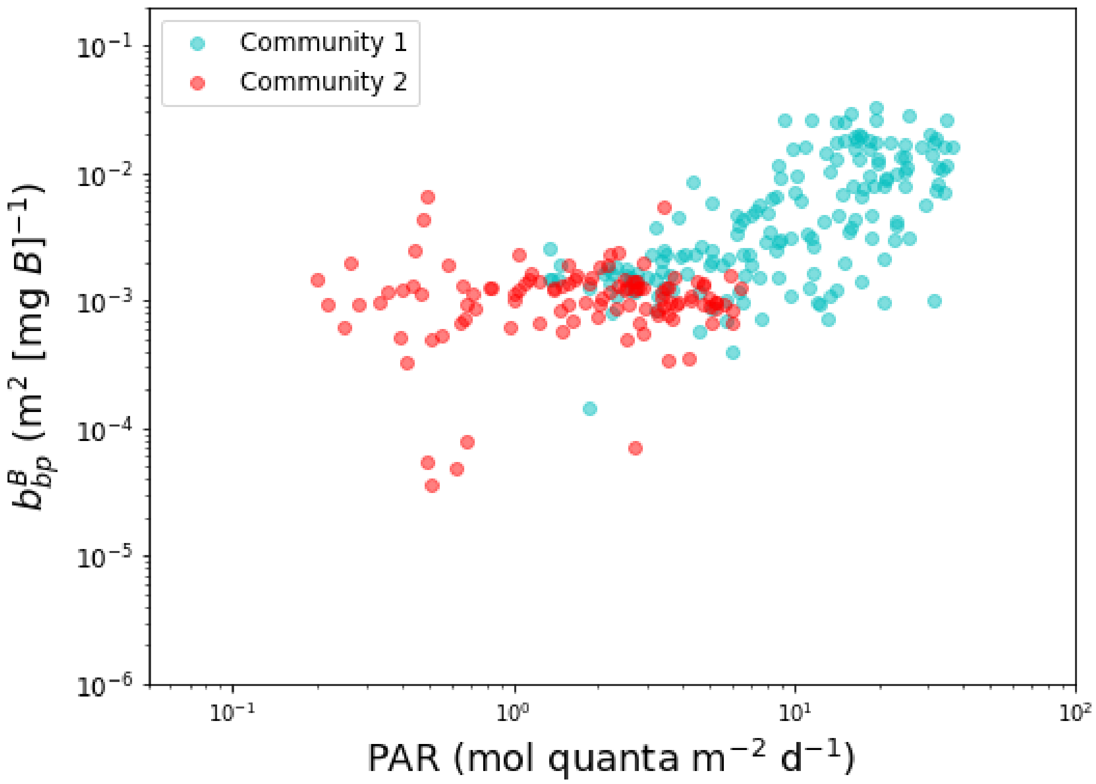

Figure S7: Relationships between the chlorophyll-specific backscattering coefficient for community 1 ($b_{bp,1}^{B}$) and the average light in the mixed-layer (cyan points), and the chlorophyll-specific backscattering coefficient for community 2 ($b_{bp,2}^{B}$) and the average light below the mixed layer and above the euphotic depth (red points).
